# Supplementary material for: Memory functions reveal structural properties of gene regulatory networks
Source: PLoS Comput Biol. 2018 Feb 22;14(2):e1006003. doi: 10.1371/journal.pcbi.1006003 (PMC5839594; doi:10.1371/journal.pcbi.1006003)
Supplement: S1 Text — In this Appendix we give the details of the projection method with linear and quadratic observables, applied to an expanded network as outlined in Sec. Nonlinear projected equations. (PDF) [file pcbi.1006003.s001.pdf]

S1 Appendix: Justification of heuristic method for quadratic observables

In this Appendix we give the details of the projection method with linear and quadratic observables, applied to an expanded network as outlined in Sec. *Nonlinear projected equations*. The expanded network is one of unary and binary reactions involving the concentrations of subnetwork proteins, $\boldsymbol{x}^{\rm s}$, of bulk proteins, $\boldsymbol{x}^{\rm b}$, and of DNA species, $\boldsymbol{x}^{\rm a}$. The mass action kinetics for this network can be put into the form of an $\boldsymbol{L}$-matrix, defined for linear and quadratic observables as in (10). We partition this into blocks according to

$$\boldsymbol{L} = \left(\begin{array}{c|c} \boldsymbol{L}^{\rm S,S} & \boldsymbol{L}^{\rm S,B} \\ \hline \boldsymbol{L}^{\rm B,S} & \boldsymbol{L}^{\rm B,B} \end{array}\right) = \left(\begin{array}{c|cc} \boldsymbol{L}^{\tilde{\rm s},\tilde{\rm s}} & \boldsymbol{L}^{\tilde{\rm s},\tilde{\rm b}} & \boldsymbol{L}^{\tilde{\rm s},\tilde{\rm a}} \\ \hline \boldsymbol{L}^{\tilde{\rm b},\tilde{\rm s}} & \boldsymbol{L}^{\tilde{\rm b},\tilde{\rm b}} & \boldsymbol{L}^{\tilde{\rm b},\tilde{\rm a}} \\ \boldsymbol{L}^{\tilde{\rm a},\tilde{\rm s}} & \boldsymbol{L}^{\tilde{\rm a},\tilde{\rm b}} & \boldsymbol{L}^{\tilde{\rm a},\tilde{\rm a}} \end{array}\right) \tag{S1.1}$$

Here $\tilde{\rm s}$ contains the "subnetwork only" observables {s} (linear) and {ss} (quadratic, like $\delta x_s \delta x_{s'}$), while $\{\tilde{\rm b}\}$ collects the slow bulk observables {b, sb, bb}. The fast bulk observables are gathered in $\{\tilde{\rm a}\}$, which contains {a, sa, ba, aa}. Note that with this partitioning of observables we have allocated all fast (DNA) species to the bulk. This is different from the approach in [21] where some fast (enzyme) species were retained in the subnetwork in order to retain more of the nonlinearities. In our case one could similarly keep in the subnetwork those DNA species that produce subnetwork proteins, but it turns out that this makes the final elimination of fast variables rather intricate and so we leave this as an option to pursue in future work.

For our GRN equations, subnetwork and bulk protein species do not interact, so the blocks $\boldsymbol{L}^{\tilde{\rm s},\tilde{\rm b}}$ and $\boldsymbol{L}^{\tilde{\rm b},\tilde{\rm s}}$ are in fact zero. This restriction is not required for our treatment, however, and direct protein-protein interactions could be included in the formalism without modification. As in the case of the linearised dynamics (Sec. *Equivalence to heuristic linearisation*), only the third column of (S1.1) is fast, *i.e.* has entries proportional to γ (plus subleading terms of order unity arising from the time derivatives of slow-fast product observables such as ba).

From (S1.1) one can obtain the rate matrix and memory functions for the projected subnetwork equations, for any finite γ. The limiting values of these quantities for $\gamma \to \infty$ can then be found from a matrix $\boldsymbol{L}_{\rm eff}$ for only the slow (protein) observables. Our aim in this Appendix is to show that this $\boldsymbol{L}_{\rm eff}$ is identical to the analogous matrix that one obtains by directly expanding the original slow (GRN) equations to second order in the protein concentrations. This then justifies the heuristic method of constructing the nonlinear rate matrix and memory functions described in Sec. *Nonlinear projected equations*.

S1.1 Generic form of notation

It will be useful to write the full time evolution equations for the expanded network in the generic form

$$\partial_t \boldsymbol{x}^{\rm l} = \boldsymbol{R}^{\rm l}(\boldsymbol{x}^{\rm l}, \boldsymbol{x}^{\rm a}) \tag{S1.2}$$

$$\gamma^{-1}\partial_t \boldsymbol{x}^{\rm a} = \boldsymbol{R}^{\rm a}(\boldsymbol{x}^{\rm l}, \boldsymbol{x}^{\rm a}) \tag{S1.3}$$

These are equations (1, 14) from the main text, or more generically (21), but we have grouped together the subnetwork and bulk concentration vectors $\boldsymbol{x}^{\rm s}$ and $\boldsymbol{x}^{\rm b}$ into a single vector of slow variables $\boldsymbol{x}^{\rm l}$ to keep the notation for the following discussion compact. The vector $\boldsymbol{x}^{\rm a}$ contains the fast variables, which in the GRN context are concentrations of DNA conformations, while γ is a fast rate parameter as before. In the limit of large γ, the fast variables are always in QSS with the slow ones so that the expanded network reduces to the thermodynamic state ensemble (in the GRN case) dynamics

$$\partial_t \boldsymbol{x}^{\rm l} = \boldsymbol{R}^{\rm l}(\boldsymbol{x}^{\rm l}, \boldsymbol{x}^{\rm a}) \tag{S1.4}$$

$$0 = \boldsymbol{R}^{\rm a}(\boldsymbol{x}^{\rm l}, \boldsymbol{x}^{\rm a}) \tag{S1.5}$$

where the second equation implicitly determines $\boldsymbol{x}^{\rm a}$ as a function of $\boldsymbol{x}^{\rm l}$. (In the main text we marked this QSS value by an asterisk; we omit this here for notational simplicity.) This is the generic form of equations (1,2) in the main text.

The matrix $\boldsymbol{L}$ is obtained in the above generic setting by expanding around a fixed point to second order to write the equations of motion as

$$\partial_t \boldsymbol{x}^{\mathrm{lT}} = \boldsymbol{x}^{\mathrm{lT}}\boldsymbol{L}^{\mathrm{ll}} + \boldsymbol{x}^{\mathrm{aT}}\boldsymbol{L}^{\mathrm{al}} + \boldsymbol{x}^{\mathrm{llT}}\boldsymbol{L}^{\mathrm{ll,l}} + \boldsymbol{x}^{\mathrm{laT}}\boldsymbol{L}^{\mathrm{la,l}} + \boldsymbol{x}^{\mathrm{aaT}}\boldsymbol{L}^{\mathrm{aa,l}} \tag{S1.6}$$

$$\gamma^{-1}\partial_t \boldsymbol{x}^{\mathrm{aT}} = \boldsymbol{x}^{\mathrm{lT}}\boldsymbol{L}^{\mathrm{la}} + \boldsymbol{x}^{\mathrm{aT}}\boldsymbol{L}^{\mathrm{aa}} + \boldsymbol{x}^{\mathrm{llT}}\boldsymbol{L}^{\mathrm{ll,a}} + \boldsymbol{x}^{\mathrm{laT}}\boldsymbol{L}^{\mathrm{la,a}} + \boldsymbol{x}^{\mathrm{aaT}}\boldsymbol{L}^{\mathrm{aa,a}} \tag{S1.7}$$

All $\boldsymbol{x}$ appearing here and below are deviations $\delta\boldsymbol{x}$ from steady state; we drop the δ to lighten the notation. The $\boldsymbol{x}^{\mathrm{ll}}$ etc are product variables – we assume the indices are ordered to avoid duplicate observables – and the $\boldsymbol{L}$ matrices contain the appropriate derivatives of the "drift" functions R at the fixed point, *e.g.* $\boldsymbol{L}^{\mathrm{ll,a}}$ has elements $L_{ll',a} = \partial_{x_l}\partial_{x_{l'}}R_a$ for $l < l'$ and $L_{ll,a} = (1/2)\partial_{x_l}\partial_{x_l}R_a$ when the two indices are equal. From the above equations then follow the evolution equations for the slow product variables $\boldsymbol{x}^{\mathrm{ll}}$ and the fast products $\boldsymbol{x}^{\mathrm{la}}$ and $\boldsymbol{x}^{\mathrm{aa}}$; see (S1.13, S1.18) below. From the product rule these equations only involve product variables on the r.h.s.; third order terms are in principle present but discarded within the second order expansion. Collecting all variables into a vector $\boldsymbol{z}$ that concatenates $\boldsymbol{x}^{\mathrm{l}}$, $\boldsymbol{x}^{\mathrm{ll}}$, $\boldsymbol{x}^{\mathrm{a}}$, $\boldsymbol{x}^{\mathrm{la}}$, $\boldsymbol{x}^{\mathrm{aa}}$ gives the time evolution equation in the form $\partial_t\boldsymbol{z}^{\mathrm{T}} = \boldsymbol{z}^{\mathrm{T}}\boldsymbol{L}$, where the matrix $\boldsymbol{L}$ has the block form (S1.1) if one restores the split of slow observables into subnetwork and bulk.

S1.2 Heuristic method

The heuristic method involves a direct expansion of the slow equations. To obtain the general form of this, one writes the dynamical equations as

$$\partial_t \boldsymbol{x}^{\mathrm{lT}} = \boldsymbol{x}^{\mathrm{lT}}\boldsymbol{L}^{\mathrm{ll}} + \boldsymbol{x}^{\mathrm{aT}}\boldsymbol{L}^{\mathrm{al}} + (\boldsymbol{x}^{\mathrm{l}}\circ\boldsymbol{x}^{\mathrm{l}})^{\mathrm{T}}\boldsymbol{L}^{\mathrm{ll,l}} + (\boldsymbol{x}^{\mathrm{l}}\circ\boldsymbol{x}^{\mathrm{a}})^{\mathrm{T}}\boldsymbol{L}^{\mathrm{la,l}} + (\boldsymbol{x}^{\mathrm{a}}\circ\boldsymbol{x}^{\mathrm{a}})^{\mathrm{T}}\boldsymbol{L}^{\mathrm{aa,l}} \tag{S1.8}$$

$$\gamma^{-1}\partial_t \boldsymbol{x}^{\mathrm{aT}} = \boldsymbol{x}^{\mathrm{lT}}\boldsymbol{L}^{\mathrm{la}} + \boldsymbol{x}^{\mathrm{aT}}\boldsymbol{L}^{\mathrm{aa}} + (\boldsymbol{x}^{\mathrm{l}}\circ\boldsymbol{x}^{\mathrm{l}})^{\mathrm{T}}\boldsymbol{L}^{\mathrm{ll,a}} + (\boldsymbol{x}^{\mathrm{l}}\circ\boldsymbol{x}^{\mathrm{a}})^{\mathrm{T}}\boldsymbol{L}^{\mathrm{la,a}} + (\boldsymbol{x}^{\mathrm{a}}\circ\boldsymbol{x}^{\mathrm{a}})^{\mathrm{T}}\boldsymbol{L}^{\mathrm{aa,a}} \tag{S1.9}$$

where the "circle" product indicates the actual products of the regular linear observables, with the same index ordering as in the projection approach (so that all the $\boldsymbol{L}$-matrices are as before). One now needs to determine $\boldsymbol{x}^{\mathrm{a}}$ by setting the r.h.s. of (S1.9) to zero, and substitute the result into (S1.8). As we are only expanding to second order in $\boldsymbol{x}^{\mathrm{l}}$, it is enough also to obtain $\boldsymbol{x}^{\mathrm{a}}$ to this order. Starting with the first order of (S1.9) one obtains

$$\boldsymbol{x}^{\mathrm{aT}} = -\boldsymbol{x}^{\mathrm{lT}}\boldsymbol{L}^{\mathrm{la}}(\boldsymbol{L}^{\mathrm{aa}})^{-1} \equiv \boldsymbol{x}_0^{\mathrm{aT}} \tag{S1.10}$$

where $\boldsymbol{x}_0^{\mathrm{a}}$ will be a convenient shorthand. (Note that the coefficient matrix $-\boldsymbol{L}^{\mathrm{la}}(\boldsymbol{L}^{\mathrm{aa}})^{-1}$ is the one we worked out in the main text below (26), in a slightly more pedestrian fashion.) All *second* order terms in (S1.9) can now be evaluated to the required accuracy by replacing $\boldsymbol{x}^{\mathrm{a}}$ by $\boldsymbol{x}_0^{\mathrm{a}}$. Solving for $\boldsymbol{x}^{\mathrm{a}}$ then gives

$$\boldsymbol{x}^{\mathrm{aT}} = \boldsymbol{x}_0^{\mathrm{aT}} - \left[(\boldsymbol{x}^{\mathrm{l}}\circ\boldsymbol{x}^{\mathrm{l}})^{\mathrm{T}}\boldsymbol{L}^{\mathrm{ll,a}} + (\boldsymbol{x}^{\mathrm{l}}\circ\boldsymbol{x}_0^{\mathrm{a}})^{\mathrm{T}}\boldsymbol{L}^{\mathrm{la,a}} + (\boldsymbol{x}_0^{\mathrm{a}}\circ\boldsymbol{x}_0^{\mathrm{a}})^{\mathrm{T}}\boldsymbol{L}^{\mathrm{aa,a}}\right](\boldsymbol{L}^{\mathrm{aa}})^{-1} \tag{S1.11}$$

Inserting into (S1.8) and dropping terms of higher than quadratic order gives the required expansion of the slow equations,

$$\begin{aligned}\partial_t \boldsymbol{x}^{\mathrm{lT}} = {} & \boldsymbol{x}^{\mathrm{lT}}\boldsymbol{L}^{\mathrm{ll}} + \boldsymbol{x}_0^{\mathrm{aT}}\boldsymbol{L}^{\mathrm{al}} + (\boldsymbol{x}^{\mathrm{l}}\circ\boldsymbol{x}^{\mathrm{l}})^{\mathrm{T}}\boldsymbol{L}^{\mathrm{ll,l}} + (\boldsymbol{x}^{\mathrm{l}}\circ\boldsymbol{x}_0^{\mathrm{a}})^{\mathrm{T}}\boldsymbol{L}^{\mathrm{la,l}} + (\boldsymbol{x}_0^{\mathrm{a}}\circ\boldsymbol{x}_0^{\mathrm{a}})^{\mathrm{T}}\boldsymbol{L}^{\mathrm{aa,l}} \\ & - \left[(\boldsymbol{x}^{\mathrm{l}}\circ\boldsymbol{x}^{\mathrm{l}})^{\mathrm{T}}\boldsymbol{L}^{\mathrm{ll,a}} + (\boldsymbol{x}^{\mathrm{l}}\circ\boldsymbol{x}_0^{\mathrm{a}})^{\mathrm{T}}\boldsymbol{L}^{\mathrm{la,a}} + (\boldsymbol{x}_0^{\mathrm{a}}\circ\boldsymbol{x}_0^{\mathrm{a}})^{\mathrm{T}}\boldsymbol{L}^{\mathrm{aa,a}}\right](\boldsymbol{L}^{\mathrm{aa}})^{-1}\boldsymbol{L}^{\mathrm{al}}\end{aligned} \tag{S1.12}$$

This then determines the $\boldsymbol{L}$-matrix for the slow variables from which rate matrix and memory functions are determined in the heuristic approach.

S1.3 Expanded network approach

As explained in the main text based on the reasoning in [21], if one writes down expressions for the rate matrix and memory functions from the full matrix $\boldsymbol{L}$ and then takes the fast rate limit $\gamma \to \infty$, the resulting rate matrix and (slow) memory function can be found from a matrix $\boldsymbol{L}_{\mathrm{eff}}$ describing only the dynamics of the slow variables $\boldsymbol{x}^{\mathrm{l}}$ and $\boldsymbol{x}^{\mathrm{ll}}$. This $\boldsymbol{L}_{\mathrm{eff}}$ is obtained by eliminating the fast variables $\boldsymbol{x}^{\mathrm{a}}$, $\boldsymbol{x}^{\mathrm{la}}$ and $\boldsymbol{x}^{\mathrm{aa}}$ using the condition that they are in QSS. These conditions are, within the projection approach, *linear* equations because product observables are treated as independent from linear observables.

What we then need to demonstrate is that this distinct elimination assigns to $\boldsymbol{x}^{\mathrm{la}}$ and $\boldsymbol{x}^{\mathrm{aa}}$ the same values as the direct expansion approach, namely $(\boldsymbol{x}^{\mathrm{l}}\circ\boldsymbol{x}_0^{\mathrm{a}})$ and $(\boldsymbol{x}_0^{\mathrm{a}}\circ\boldsymbol{x}_0^{\mathrm{a}})$. Once this is shown, it follows that the linear fast variables $\boldsymbol{x}^{\mathrm{a}}$ are eliminated in the same way in the two approaches, because the same quadratic fast variables are substituted into the relevant equations (S1.7,S1.9). Thus *all* fast variables are eliminated in the same way from the time evolution equation for the slow variables, to the quadratic order we consider here. As the slow $\boldsymbol{L}$-matrix in the heuristic approach and the $\boldsymbol{L}_{\mathrm{eff}}$ in the expanded networm method are both obtained from this time evolution equation for the slow variables, they are therefore equal as we want to show.

S1.3.1 Elimination of $\boldsymbol{x}^{\mathrm{la}}$

Using the product rule of differentiation, the equations of motion for the $\boldsymbol{x}^{\mathrm{la}}$ are

$$\partial_t \boldsymbol{x}^{\mathrm{laT}} = \gamma((\boldsymbol{x}^{\mathrm{lT}} \circ \boldsymbol{x}^{\mathrm{lT}}\boldsymbol{L}^{\mathrm{la}})) + \gamma((\boldsymbol{x}^{\mathrm{lT}} \circ \boldsymbol{x}^{\mathrm{aT}}\boldsymbol{L}^{\mathrm{aa}})) + ((\boldsymbol{x}^{\mathrm{lT}}\boldsymbol{L}^{\mathrm{ll}} \circ \boldsymbol{x}^{\mathrm{aT}})) + ((\boldsymbol{x}^{\mathrm{aT}}\boldsymbol{L}^{\mathrm{al}} \circ \boldsymbol{x}^{\mathrm{aT}})) \tag{S1.13}$$

Here the double brackets on the right indicate that after the circle products are taken the real products have to be replaced by product variables, to remain within the projection framework.

We want to eliminate the $\boldsymbol{x}^{\mathrm{la}}$ from the condition that the r.h.s. vanishes. Fortunately for large γ the first two terms, which stem from the time evolution of $\boldsymbol{x}^{\mathrm{a}}$, dominate; the last two become negligible in comparison. Thus one has to solve

$$((\boldsymbol{x}^{\mathrm{lT}} \circ \boldsymbol{x}^{\mathrm{lT}}\boldsymbol{L}^{\mathrm{la}})) + ((\boldsymbol{x}^{\mathrm{lT}} \circ \boldsymbol{x}^{\mathrm{aT}}\boldsymbol{L}^{\mathrm{aa}})) = 0 \tag{S1.14}$$

From the structure of this one sees that the $\boldsymbol{x}^{\mathrm{l}}$ only act as "spectators", while considering the second factors one has to solve the same equation as at linear order. The solution is therefore expected to be $\boldsymbol{x}^{\mathrm{laT}} = ((\boldsymbol{x}^{\mathrm{lT}} \circ \boldsymbol{x}_0^{\mathrm{aT}}))$ as we want to show.

To see this in more detail we write out (S1.14) in components:

$$\sum_{l'} x_{(ll')} L_{l'a} + \sum_{a'} x_{la'} L_{a'a} = 0 \tag{S1.15}$$

Here we have written $x_{(ll')}$ to indicate that the indices are to be taken as ordered, *i.e.* $x_{(ll')} = x_{ll'}$ if $l \le l'$ and $x_{(ll')} = x_{l'l}$ otherwise. The proposed solution is

$$x_{la} = ((\boldsymbol{x}^{\mathrm{lT}} \circ \boldsymbol{x}_0^{\mathrm{aT}}))_{la} = -\sum_{l'a'} x_{(ll')} L_{l'a'} (\boldsymbol{L}^{\mathrm{aa}})^{-1}_{a'a} \tag{S1.16}$$

This does solve (S1.15) because

$$\sum_{a''} x_{la''} L_{a''a} = -\sum_{l'a'a''} x_{(ll')} L_{l'a'} (\boldsymbol{L}^{\mathrm{aa}})^{-1}_{a'a''} L_{a''a} = -\sum_{l'} x_{(ll')} L_{l'a} \tag{S1.17}$$

S1.4 Elimination of $\boldsymbol{x}^{\mathrm{aa}}$

We proceed again using the product rule of differentiation, which gives as the equations of motion for the $\boldsymbol{x}^{\mathrm{aa}}$

$$\partial_t \boldsymbol{x}^{\mathrm{aaT}} = \gamma((\boldsymbol{x}^{\mathrm{lT}}\boldsymbol{L}^{\mathrm{la}} \circ \boldsymbol{x}^{\mathrm{aT}})) + \gamma((\boldsymbol{x}^{\mathrm{aT}}\boldsymbol{L}^{\mathrm{aa}} \circ \boldsymbol{x}^{\mathrm{aT}})) + \gamma((\boldsymbol{x}^{\mathrm{aT}} \circ \boldsymbol{x}^{\mathrm{lT}}\boldsymbol{L}^{\mathrm{la}})) + \gamma((\boldsymbol{x}^{\mathrm{aT}} \circ \boldsymbol{x}^{\mathrm{aT}}\boldsymbol{L}^{\mathrm{aa}})) \tag{S1.18}$$

Here all terms contribute for large γ, but one sees that in the first two the right factor of $\boldsymbol{x}^{\mathrm{a}}$ is again a "spectator" and similarly with the left factor for the last two terms. Accordingly one can show that the proposed solution, which is $\boldsymbol{x}^{\mathrm{aaT}} = ((\boldsymbol{x}_0^{\mathrm{aT}} \circ \boldsymbol{x}_0^{\mathrm{aT}}))$, ensures that each pair of terms vanishes separately.

For explicit calculation it is again useful to write out components. The aa' component of the first two terms of (S1.18), without the overall factor of γ, reads

$$\sum_{l'} x_{l'a'} L_{l'a} + \sum_{a''} x_{(a'a'')} L_{a''a} \tag{S1.19}$$

The proposed solution is

$$x_{aa'} = ((\boldsymbol{x}_0^{\mathrm{aT}} \circ \boldsymbol{x}_0^{\mathrm{aT}}))_{aa'} = \sum_{l_1 l_2 a_1 a_2} x_{(l_1 l_2)} L_{l_1 a_1} (\boldsymbol{L}^{\mathrm{aa}})^{-1}_{a_1 a} L_{l_2 a_2} (\boldsymbol{L}^{\mathrm{aa}})^{-1}_{a_2 a'} \tag{S1.20}$$

Substituting this and the solution (S1.16) for x_{la} turns (S1.19) into

$$-\sum_{l'l''a''} x_{(l'l'')} L_{l''a''} (\boldsymbol{L}^{\mathrm{aa}})^{-1}_{a''a'} L_{l'a} + \sum_{a'' l_1 l_2 a_1 a_2} x_{(l_1 l_2)} L_{l_1 a_1} (\boldsymbol{L}^{\mathrm{aa}})^{-1}_{a_1 a'} L_{l_2 a_2} (\boldsymbol{L}^{\mathrm{aa}})^{-1}_{a_2 a''} L_{a''a} \tag{S1.21}$$

The last factors in the second term again cancel, reducing it to

$$\sum_{l_1 l_2 a_1} x_{(l_1 l_2)} L_{l_1 a_1} (\boldsymbol{L}^{\mathrm{aa}})^{-1}_{a_1 a'} L_{l_2 a} \tag{S1.22}$$

After a relabelling of summation indices this is identical to the first term. This means that (S1.19) vanishes, *i.e.* the first two terms on the r.h.s. of (S1.18) cancel. Similarly the last two terms vanish, showing that (S1.20) is the correct QSS assignment of the $\boldsymbol{x}^{\mathrm{aa}}$.

S1.4.1 Equations of motion for $\boldsymbol{x}^{\mathrm{ll}}$

Above we have shown that the direct and the projection elimination procedures give the same time evolution equation for $\boldsymbol{x}^{\mathrm{l}}$. The same can then also be checked straightforwardly for the product variables $(\boldsymbol{x}^{\mathrm{l}} \circ \boldsymbol{x}^{\mathrm{l}})$ and their projection analogues $\boldsymbol{x}^{\mathrm{ll}}$. By analogy with (S1.13), the latter evolve in time according to

$$\partial_t \boldsymbol{x}^{\mathrm{llT}} = ((\boldsymbol{x}^{\mathrm{lT}} \circ \boldsymbol{x}^{\mathrm{lT}} \boldsymbol{L}^{\mathrm{ll}})) + ((\boldsymbol{x}^{\mathrm{lT}} \circ \boldsymbol{x}^{\mathrm{aT}} \boldsymbol{L}^{\mathrm{al}})) + ((\boldsymbol{x}^{\mathrm{lT}} \boldsymbol{L}^{\mathrm{ll}} \circ \boldsymbol{x}^{\mathrm{lT}})) + ((\boldsymbol{x}^{\mathrm{aT}} \boldsymbol{L}^{\mathrm{al}} \circ \boldsymbol{x}^{\mathrm{lT}})) \tag{S1.23}$$

The real products obey the same equation, just written differently:

$$\partial_t (\boldsymbol{x}^{\mathrm{l}} \circ \boldsymbol{x}^{\mathrm{l}})^{\mathrm{T}} = (\boldsymbol{x}^{\mathrm{lT}} \circ \boldsymbol{x}^{\mathrm{lT}} \boldsymbol{L}^{\mathrm{ll}}) + (\boldsymbol{x}^{\mathrm{lT}} \circ \boldsymbol{x}^{\mathrm{aT}} \boldsymbol{L}^{\mathrm{al}}) + (\boldsymbol{x}^{\mathrm{lT}} \boldsymbol{L}^{\mathrm{ll}} \circ \boldsymbol{x}^{\mathrm{lT}}) + (\boldsymbol{x}^{\mathrm{aT}} \boldsymbol{L}^{\mathrm{al}} \circ \boldsymbol{x}^{\mathrm{lT}}) \tag{S1.24}$$

From both, the fast products $(\boldsymbol{x}^{\mathrm{l}} \circ \boldsymbol{x}^{\mathrm{a}})$ (respectively $\boldsymbol{x}^{\mathrm{la}}$) and $(\boldsymbol{x}^{\mathrm{a}} \circ \boldsymbol{x}^{\mathrm{a}})$ (respectively $\boldsymbol{x}^{\mathrm{aa}}$) then need to be eliminated. As we have already established that these eliminations are identical, also the resulting equations for the ll-product variables must be identical.
