# Supplementary material for: Memory functions reveal structural properties of gene regulatory networks
Source: PLoS Comput Biol. 2018 Feb 22;14(2):e1006003. doi: 10.1371/journal.pcbi.1006003 (PMC5839594; doi:10.1371/journal.pcbi.1006003)
Supplement: S2 Text — In this Appendix we give additional detail of the wide variety of effects that memory functions contribute to the behaviour of a system. Specifically, we explore the effect of the contributions from nonlinear memory terms and provide an alternative visualisation using a first order time expansion of the effective drift of the system. (PDF) [file pcbi.1006003.s002.pdf]

S2 Appendix: Further analysis of memory effects

In this Appendix we give additional detail of the wide variety of effects that memory functions contribute to the behaviour of a system. Specifically, we explore the effect of the contributions from nonlinear memory terms and provide an alternative visualisation using a first order time expansion of the effective drift of the system.

S2.1 Nonlinear memory effects

In Fig. S1 we show trajectory plots comparing the predictions without memory (left column) to those with linear and linear+quadratic memory (middle columns) and to the dynamics of the full network. Focussing directly onto the description with all memory terms included, we can gain insight into what drives the distinct dynamics of Fig. S1C,G by analysing the respective nonlinear memory functions, specifically their amplitude (value at $\Delta t = 0$) as shown in Fig. S2A-D. We note that in most cases the memory contributions arise via Irx3. (In the case of memory of Olig2 this is the only possibility in any case.) A clear exception to this is the case of nonlinear Nkx2.2 memory to itself (Fig. S2A) where the memory is dominated ventrally by the Pax6 channel and dorsally by the Irx3 channel. These memory functions also change with time difference, but it turns out that they decay on similar timescales so that the memory amplitude gives a reasonable characterization of their strength. We observe that Olig2 does not produce memory effects that are substantial enough to change the dynamics (Fig. S2B,D): the dominant nonlinear memory effects come from Nkx2.2 (Fig. S2A,C). We see further that Nkx2.2 represses both itself (negative nonlinear memory amplitude, Fig. S2A) and, even more strongly, Olig2 (Fig. S2C). These two terms combined mean that at high levels of Nkx2.2, the memory functions will impede Olig2 from increasing as quickly as it would in cases without nonlinear memory, while simultaneously decreasing the concentration of Nkx2.2. Both effects are consistent with what we observe in Fig. S1C, D.

In the p2 domain the memoryless and linear memory predictions for trajectories are quite close, with levels of Nkx2.2 and Olig2 decreasing on similar timescales towards the steady state (Fig. S1E–H). Nonetheless, plots showing the norm of the effective drift indicate the trend that memory pushes the system more quickly towards low Nkx2.2 (Fig. S3D,E, see below). Including the nonlinear memory then further enhances this tendency: the levels of Nkx2.2 drop more rapidly than Olig2 on the approach to the steady state (Fig. S1G,H & Fig. S3F). The behaviour is not identical to but mimics that of the full thermodynamic model: the nonlinear memory effects contribute qualitative information about the behaviour of the system further away from the steady state. This points to memory terms being important for first reducing levels of Nkx2.2 before reducing Olig2 levels.

Understanding why this is the case requires a more detailed analysis of the memory effects. The memory amplitudes show that Nkx2.2 is not subject to substantial memory effects (Fig. S2E,F) and the largest memory effects act on Olig2 (Fig. S2G,H). At first glance, the latter memory amplitudes suggests that Nkx2.2 substantially represses Olig2 while Olig2 activates itself to a lesser degree. However, on closer inspection of the temporal dependence of the memory functions, the repression by Nkx2.2 is a short pulse that for larger Δt turns into an activation of Olig2 (Fig. S1F). Meanwhile, the Olig2 self-activation is a sustained signal that lasts much longer than the repression by Nkx2.2. When taken together, these two memory terms (combined with cross terms, not shown) promote activation of Olig2 from high levels of Nkx2.2 even after a substantial time difference has elapsed, of the order of the decay time of the linear memory functions. This is consistent with what we observe in Fig. S1G,H: the initial high levels of Nkx2.2 lead to Olig2 levels being sustained via the memory, while Nkx2.2 decays in a way almost unaffected by memory. Eventually Olig2 also decays, once enough time has passed for the nonlinear memory effects to fade away.

S2.2 Calculation of effective drift in the presence of memory

It is useful to be able to visualize the effects of memory terms in the projected equations in terms of an effective drift. This is possible at least perturbatively for short times, as we now show. We illustrate the method for the linearized dynamics, where the projected equation (3) without the random force read in vector form:

$$\partial_t \boldsymbol{x}^{\mathrm{T}} = \boldsymbol{x}(t)^{\mathrm{T}} \boldsymbol{\Omega} + \int_0^t dt' \boldsymbol{x}(t)^{\mathrm{T}} \boldsymbol{M}(t - t') \tag{S2.1}$$

For small times t, the memory function can be treated as approximately constant so that one obtains in an expansion to first order in t:

$$\int_0^t dt' \boldsymbol{x}(t)^{\mathrm{T}} \boldsymbol{M}(t-t') \simeq t\, \boldsymbol{x}(t)^{\mathrm{T}} \boldsymbol{M}(0) \tag{S2.2}$$

Combining with the rate matrix term gives the effective drift

$$\partial_t \boldsymbol{x}^{\mathrm{T}} = \boldsymbol{x}(t)^{\mathrm{T}} [\boldsymbol{\Omega} + t\boldsymbol{M}(0)] \tag{S2.3}$$

On the right hand side one has a function of the current concentrations only, as announced. Given that this is a linear expansion in t we refrain from exploring the system too far away from $t = 0$. Higher order expansions in t could be performed yet the first order is enough to demonstrate the qualitative contributions of the memory terms.

We illustrate the approach in Fig. S3A-F with contour plots of the norm of the effective drift vector on the r.h.s. of (S2.3), for time $t = 0.8$. Bearing in mind that the system will pass quickly through regions where the drift is high and spend most of its time in regions where it is low, these plots confirm the effects seen in the trajectory plots: inclusion of the memory terms causes the low-drift region to shift to lower Nkx2.2 concentrations, *i.e.* the system will more rapidly reduce Nkx2.2 and then spend more time increasing Olig2 at small Nkx2.2.
