# Supplementary material for: Memory functions reveal structural properties of gene regulatory networks
Source: PLoS Comput Biol. 2018 Feb 22;14(2):e1006003. doi: 10.1371/journal.pcbi.1006003 (PMC5839594; doi:10.1371/journal.pcbi.1006003)
Supplement: S3 Text — Equations and parameters used in model of neural tube patterning network. (PDF) [file pcbi.1006003.s003.pdf]

S3 Appendix: Model details

The full network equations for the neural tube application in Sec. *Application to neural tube network* are of the form (1, 2) and taken from [24]:

$$\partial_t[\mathrm{P}] = \alpha_{\mathrm{P}} \frac{w_{\mathrm{P}}}{w_{\mathrm{P}} + (1 + w_{\mathrm{PO}}[\mathrm{O}])^2 (1 + w_{\mathrm{PN}}[\mathrm{N}])^2} - \beta_{\mathrm{P}}[\mathrm{P}]$$

$$\partial_t[\mathrm{O}] = \alpha_{\mathrm{O}} \frac{w_{\mathrm{O}}(1 + w_{\mathrm{OS}}[\mathrm{S}])}{w_{\mathrm{O}}(1 + w_{\mathrm{OS}}[\mathrm{S}]) + (1 + w_{\mathrm{ON}}[\mathrm{N}])^2 (1 + w_{\mathrm{OI}}[\mathrm{I}])^2} - \beta_{\mathrm{O}}[\mathrm{O}]$$

$$\partial_t[\mathrm{N}] = \alpha_{\mathrm{N}} \frac{w_{\mathrm{N}}(1 + w_{\mathrm{NS}}[\mathrm{S}])}{w_{\mathrm{N}}(1 + w_{\mathrm{NS}}[\mathrm{S}]) + (1 + w_{\mathrm{NP}}[\mathrm{P}])^2 (1 + w_{\mathrm{NO}}[\mathrm{O}])^2 (1 + w_{\mathrm{NI}}[\mathrm{I}])^2} - \beta_{\mathrm{N}}[\mathrm{N}]$$

$$\partial_t[\mathrm{I}] = \alpha_{\mathrm{I}} \frac{w_{\mathrm{I}}}{w_{\mathrm{I}} + (1 + w_{\mathrm{IO}}[\mathrm{O}])^2 (1 + \mathrm{w}_{\mathrm{IN}}[\mathrm{N}])^2} - \beta_{\mathrm{I}}[\mathrm{I}]$$

Here [S] is the level of Sonic Hedgehog signaling - the net amount of Gli activity, see [24] for details. This is taken to be an exponential gradient, $[\mathrm{S}] = e^{-s/0.15}$ where $s \in [0, 1.2]$ labels the neural tube position. The remaining symbols in brackets indicate the concentration of the TFs. Parameters used are $\alpha_{\mathrm{P}} = 2$, $\alpha_{\mathrm{O}} = 2$, $\alpha_{\mathrm{N}} = 2$, $\alpha_{\mathrm{I}} = 2$, $\beta_{\mathrm{P}} = 2$, $\beta_{\mathrm{O}} = 2$, $\beta_{\mathrm{N}} = 2$, $\beta_{\mathrm{I}} = 2$, $w_{\mathrm{PO}} = 1.9$, $w_{\mathrm{PN}} = 26.7$, $w_{\mathrm{P}} = 3.84$, $w_{\mathrm{ON}} = 60.6$, $w_{\mathrm{OI}} = 28.4$, $w_{\mathrm{OS}} = 180$, $w_{\mathrm{O}} = 38.24$, $w_{\mathrm{NP}} = 4.8$, $w_{\mathrm{NO}} = 27.1$, $w_{\mathrm{NI}} = 47.1$, $w_{\mathrm{NS}} = 373$, $w_{\mathrm{N}} = 21.92$, $w_{\mathrm{IO}} = 58.8$, $w_{\mathrm{IN}} = 76.2$, $w_{\mathrm{I}} = 18.72$.
