# Supplementary figures and images for: Memory functions reveal structural properties of gene regulatory networks
Source: PLoS Comput Biol. 2018 Feb 22;14(2):e1006003. doi: 10.1371/journal.pcbi.1006003 (PMC5839594; doi:10.1371/journal.pcbi.1006003)

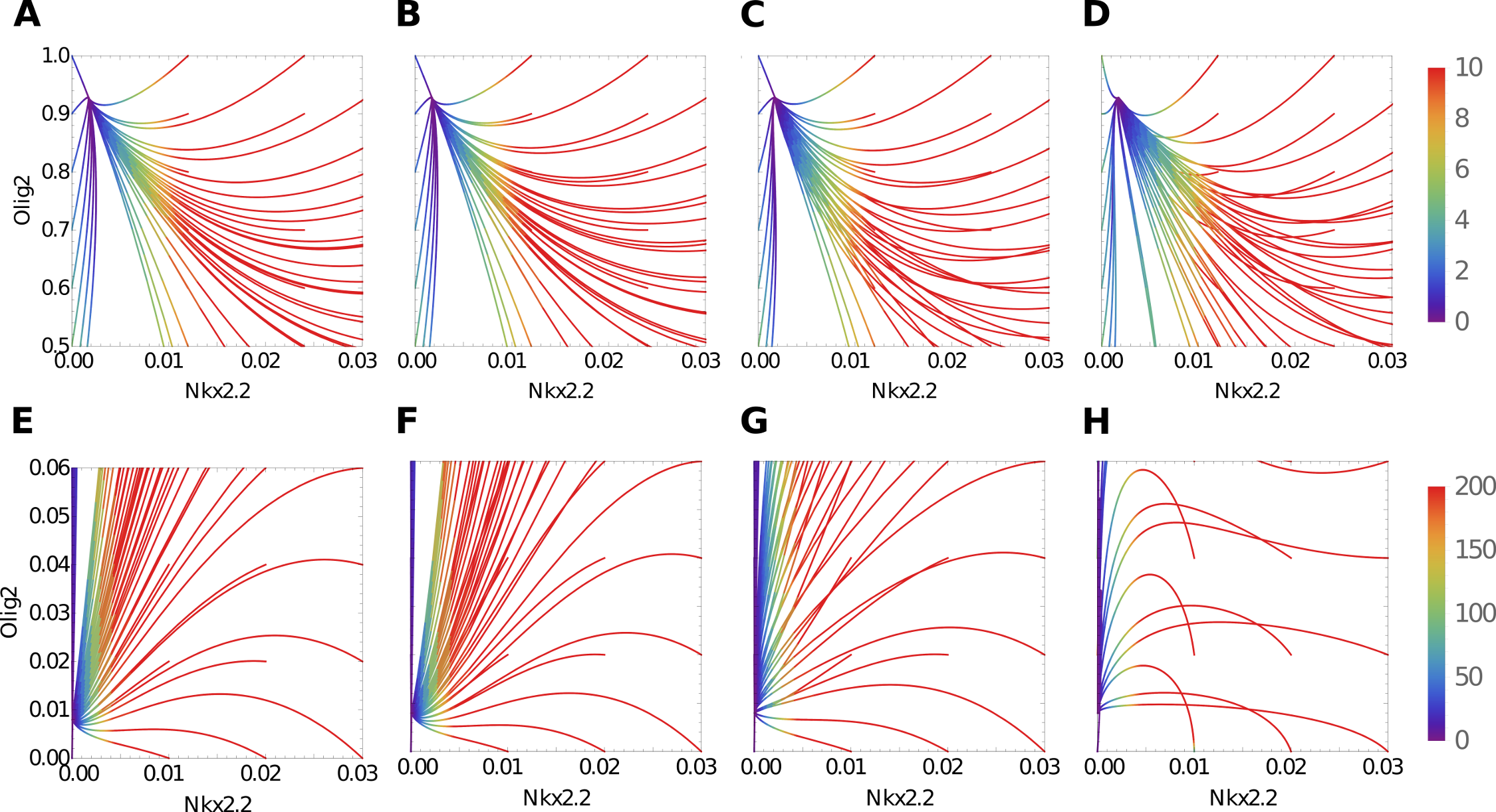

Supplement: S1 Fig — Trajectories approaching a high Olig2 state in the pMN domain from a range of initial conditions, with no memory (A), with only linear memory (B), with linear and nonlinear memory (C), and the full dynamics (D). Trajectories approaching a low Olig2, low Nkx2.2 state in the p2 domain from different initial conditions, with no memory (E), with only linear memory (F), with linear and nonlinear memory (G), and the full dynamics (H). All figures are parametric plots, showing Nkx2.2 and Olig2 concentrations on the x- and y-axis, respectively, with time as curve parameter. The trajectories are coloured to represent the norm of the drift vector of the system as indicated by the colour scale; high values indicate the system is evolving quickly while the opposite is true for low values. Scalebars on the right apply to the corresponding row. Path crossing can be seen in (C–D) & (G–H), illustrating the importance of nonlinear memory terms to reproduce qualitative features of the full thermodynamic equations. (TIF) [file pcbi.1006003.s004.tif]

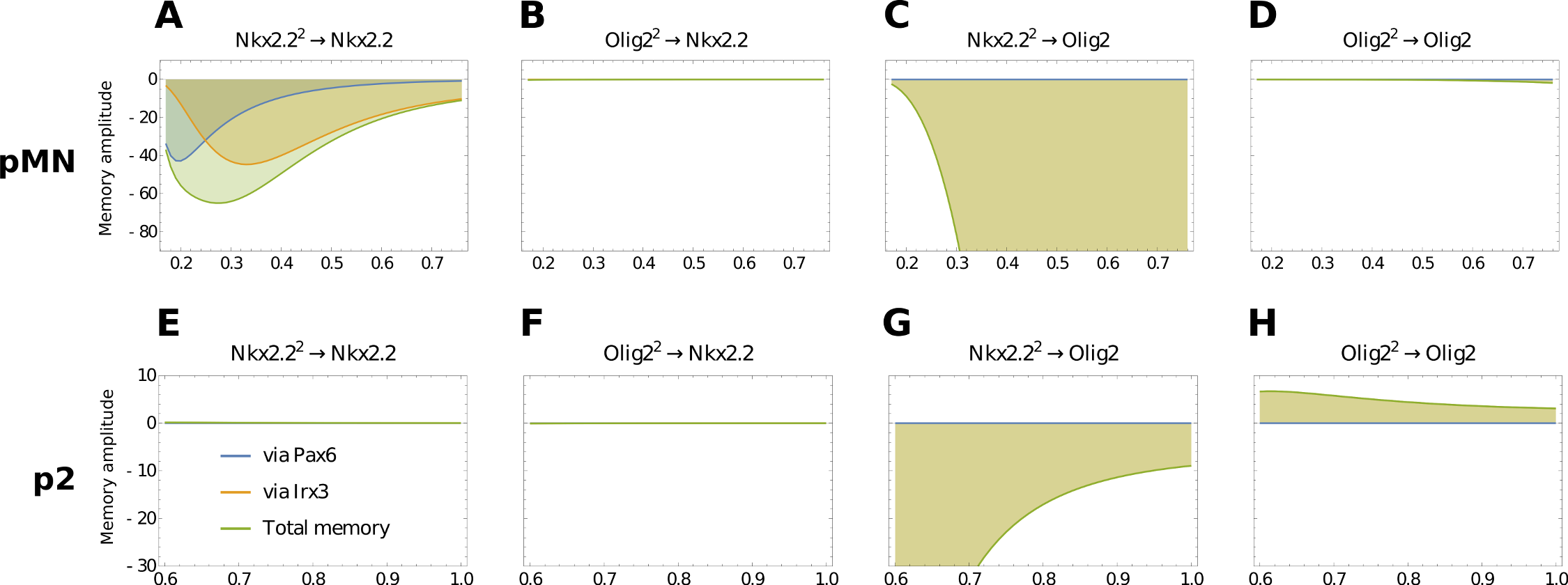

Supplement: S2 Fig — (A-D) Nonlinear memory amplitudes across neural tube positions within the pMN domain. The plot titles indicate the type of memory, e.g. (C) shows memory of (past) Nkx2.2 squared fluctuation on Olig2. Memory effects of past Olig2-fluctuations in the pMN domain are negligible. (E-H) Nonlinear memory amplitudes across neural tube positions within the p2 domain. Nkx2.2 receives very little quadratic memory influence in this domain. The x-axis represents neural tube position in all plots. Blue and yellow lines indicate the decomposition into Pax6 and Irx3 channels, while green lines indicate the total memory. (TIF) [file pcbi.1006003.s005.tif]

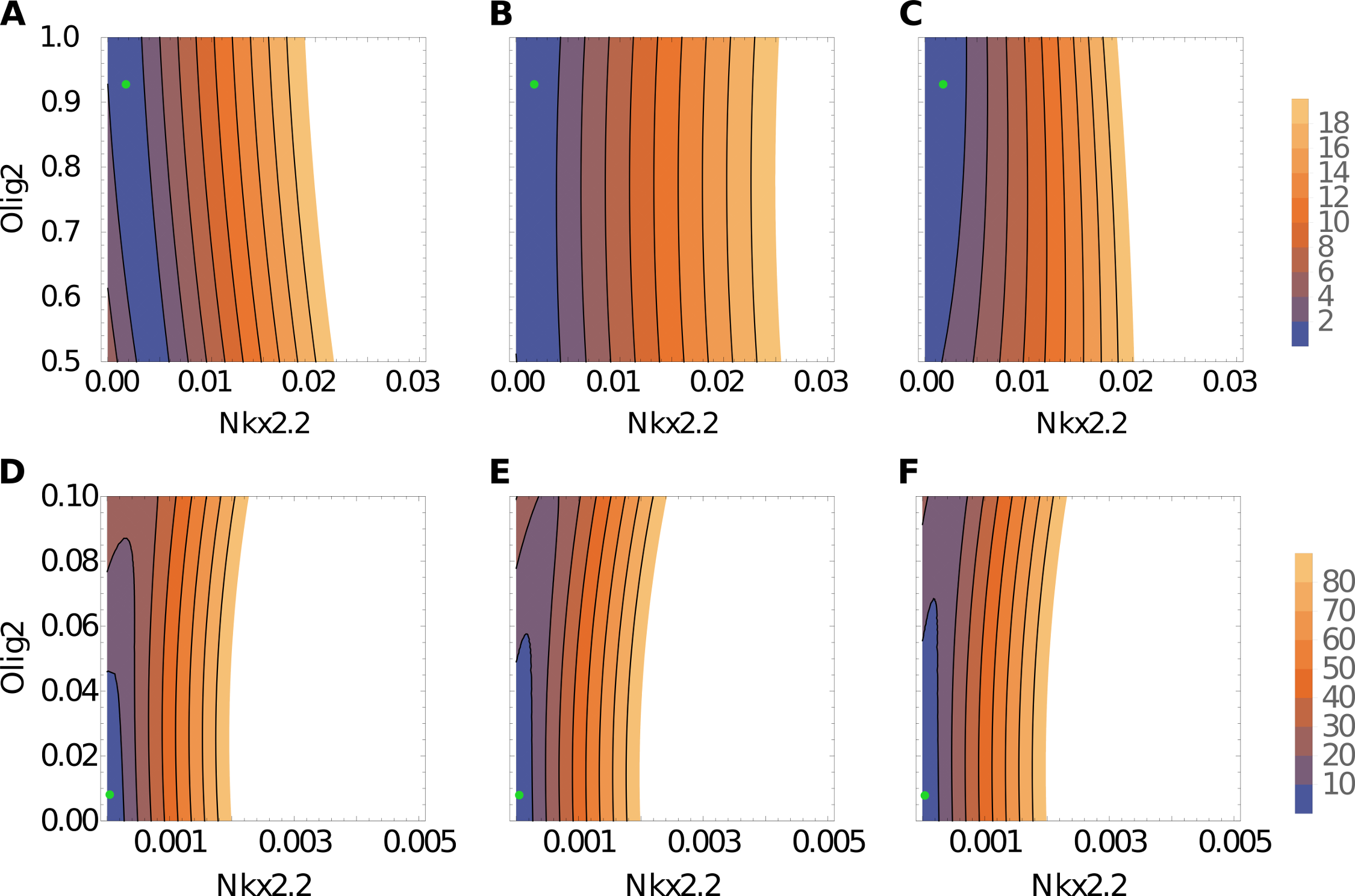

Supplement: S3 Fig — Colour map and contour plots of the norm of the effective drift, in conditions identical to those in S1A–S1C Fig for the first row (pMN domain) and S1E–S1G Fig (p2 domain) for the second row. (A,D) Memoryless drift, (B,E) drift with linear memory, (C,F) drift with linear and nonlinear memory. The scalebar on the right applies to each entire row. Effective drift norms have been calculated for time t = 0.8. (TIF) [file pcbi.1006003.s006.tif]
